# The long-term trend in utilization of traditional Chinese medicine and associated factors among older people in Taiwan

**DOI:** 10.1371/journal.pone.0302658

**Published:** 2024-05-08

**Authors:** Chien-Jung Huang, Chuen-Chau Chang, Ta-Liang Chen, Chun-Chieh Yeh, Jaung-Geng Lin, Chieh-Hsing Liu, Chien-Chang Liao

**Affiliations:** 1 Department of Health Promotion and Health Education, National Taiwan Normal University, Taipei, Taiwan; 2 Department of Anesthesiology, Taipei Medical University Hospital, Taipei, Taiwan; 3 Anesthesiology and Health Policy Research Center, Taipei Medical University Hospital, Taipei, Taiwan; 4 Department of Anesthesiology, School of Medicine, College of Medicine, Taipei Medical University, Taipei, Taiwan; 5 Department of Anesthesiology, Wan Fang Hospital, Taipei Medical University, Taipei, Taiwan; 6 Department of Surgery, China Medical University Hospital, Taichung, Taiwan; 7 Department of Surgery, University of Illinois, Chicago, IL, United States of America; 8 School of Chinese Medicine, College of Chinese Medicine, China Medical University, Taichung, Taiwan; 9 Research Center of Big Data and Meta-Analysis, Wan Fang Hospital, Taipei Medical University, Taipei, Taiwan; Baotou Medical College, MONGOLIA

## Abstract

**Background:**

Both the size of the older population and the use of complementary and alternative medicine are increasing worldwide. This study evaluated the long-term trend in utilization of traditional Chinese medicine (TCM) and associated factors among older people in Taiwan.

**Methods:**

Using the database of population-based interview surveys, we evaluated the one-month prevalence of TCM use among 13,945 older people aged over 65 years from 2001–2017. The sociodemographic status and medical comorbidities of older people who did and did not use TCM were compared by calculating adjusted odds ratios (ORs) and 95% confidence intervals (CIs) in the multiple logistic regressions.

**Results:**

The one-month prevalence of TCM use increased from 5.5% in 2001 to 9.1% in 2017 among older people in Taiwan. Overall, 7.3% of older people had used TCM within the previous month. People with a history of heart disease (OR 1.62, 95% CI 1.24–2.12), use of folk therapy (OR 3.16, 95% CI 2.00–4.99), and purchase of non-prescribed Chinese herbal medicine (OR 2.08, 95% CI 1.48–2.91) were more likely to use TCM than the comparison group. However, age ≥80 years (OR 0.48, 95% CI 0.31–0.72) and previous hospitalization (OR 0.59, 95% CI 0.41–0.85) were associated with the reduced use of TCM.

**Conclusion:**

From 2001–2017, the use of TCM increased in the older population in Taiwan. The use of folk medicine and purchase of non-prescribed Chinese herbal medicine were significant predictors for the use of TCM.

## Introduction

The rapid increase in the population and related medical care of older people are major concerns that affect all countries [1–3]. It is estimated that the population of people older than 65 years is projected to rise from 771 million in 2022 to 1.6 billion in 2050 worldwide [4]. A high annual medical expenditure of older people can be expected because the per capita lifetime expenditure of health care is $316,600 and nearly half during the senior years [5]. Undoubtedly, biochemical medicine, also called conventional medicine or Western medicine, is the most commonly used type of medical care. However, the increasing use of complementary and alternative medicine (CAM) has attracted attention, and medical pluralism is a trend worldwide [6].

CAM is the term for medical products and practices that are not part of standard medical care that became popular worldwide in the past 20 years [7]. It has been reported that the one-year prevalence of CAM use among American, Asian-American, and European older people is 27%-62%, 48–76%, and 33.5%, respectively [8–11]. Traditional Chinese medicine (TCM) is a complete CAM healing system and is widely used in Taiwan, China, Korea, Japan, and other Asian countries [12–17]. In the general population in Taiwan, the prevalence rates of TCM use for one month, one year, and six years were reported as 10.4%, 28.4%, and 62.5%, respectively [14–16]. In a cross-sectional study in Japan, 71.5% of interviewees requested CAM practice at hospitals, and most of them accepted TCM [17]. The therapeutic effect of TCM has been confirmed, and it is commonly used in patients with cardiovascular diseases, kidney disease, stroke, cancer, and other diseases [18, 19]. Previous studies have also investigated the beneficial effects of TCM on patients with COVID-19. Given the comprehensive nature of TCM, it is a well-suited choice for utilization among elderly individuals.

In Shanghai, the lifetime prevalence of the use of TCM among people aged 50 years and older was estimated to be as high as 50% [12]. Among Korean people who used traditional Korean medicine (similar to TCM) in the past year, 41.8% of them were older people aged over 65 years [13]. In Taiwan, the 6-year prevalence (1996–2001) of TCM use among older people aged over 61 years was as high as 61% [20]. Another report also showed that 48% of elderly patients aged 65 years and older had used TCM at least once from 2005–2009 in Taiwan [21]. However, no studies have focused on TCM use among older people after 2010.

In Taiwan’s health care system, there are two types of medical doctor licenses: one for Western medicine physicians and another for TCM physicians. Both medical care systems are covered by National Health Insurance, and people do not need to pay much money when seeking medical care by Western medicine (WM) or TCM physicians in Taiwan. Although the utilization rate of TCM among older people has been studied in previous reports, information regarding recent full-spectrum utilization of TCM is limited. Using population-based data from an interview survey, the purpose of this study was to evaluate the prevalence, frequency, and associated factors of TCM use among people aged 65 years and older in Taiwan.

## Methods

### Data source

The Health Promotion Administration, Ministry of Health and Welfare in Taiwan conducted the National Health Interview Survey (NHIS) using face-to-face questionnaire interviews in 2001, 2005, 2009, 2013, and 2017. Taiwan is an island of approximately 23 million people, 18% of whom are older than 65 years. These five NHISs (including 2522 participants in 2001, 2729 participants in 2005, 2703 participants in 2009, 2820 participants in 2013, and 3171 participants in 2017) consisted of a representative sample including 13945 interviewees aged 65 years from the noninstitutionalized population. These five NHISs were independent and they were conducted in different calendar year all over Taiwan. There were no duplicate participants in these five NHISs. Each NHIS interview was performed in the subject’s home, and the response rate was 80.6%. Details of the NHIS were described clearly in previous studies [6, 15, 22, 23].

At the initial interview during the NHIS, the participants were asked for permission for access to the interview database for research purposes, and all study participants signed an informed consent form. To protect personal privacy, the electronic database was encoded, and the identifiers of participants were scrambled prior to further academic access for research. Written informed consent was obtained from eligible NHIS participants, and our study was evaluated and approved by the Joint Institutional Review Board of Taipei Medical University (TMU-JIRB-202303013; TMU-JIRB-202301004).

Information about the prevalence and frequency of TCM and Western medicine use was drawn from the data of the 2001, 2005, 2009, 2013, and 2017 NHISs. The core question in this study was “Did you seek traditional Chinese medicine for yourself with a physician’s prescription or advice in the past month?” Age, sex, education, occupation, family income, ethnicity, religion and marital status, history of disease (heart disease, hypertension, pulmonary disease, diabetes, hyperlipidemia, stroke, liver disease, and kidney disease), use of medical resources (emergency services, hospitalization, and influenza vaccination), use of folk medicine, purchase of non-prescribed Chinese herbal medicine (CHM), smoking, and alcohol consumption were derived from the NHIS data.

### Definition and criteria

The NHIS is a cross-sectional survey that was conducted by Health Promotion Administration, Ministry of Health and Welfare. The information of marital status, history of disease, smoking, alcohol consumption, emergency care, inpatient care, influenza vaccination, use of folk medicine, and purchase of non-prescribed CHM were defined as within recent 12 months before the face-to-face questionnaire interview.

In general, CAM is the term for medical products and practices that are not part of standard medical care (that also called Western medicine, conventional medicine, and biomedical medicine). Types of CAM included mind-body therapies (such as meditation, biofeedback, hypnosis, yoga, tai chi, imagery, and creative outlets), biologically based practices (such as vitamins, dietary supplements, botanicals, and special foods or diets), manipulative and body-based practices (such as massage therapy, chiropractic therapy, and reflexology), energy healing (such as reiki and therapeutic touch), and whole medical systems (such as TCM, ayurvedic medicine, and naturopathic medicine).

In Taiwan, TCM includes CHM, acupuncture, moxibustion, bone reduction, traditional trauma treatment, traditional dislocation treatment, traditional fracture treatment, tui-na, ba-guan, and other therapies. Like Western medicine, TCM has also been covered by National Health Insurance since 1995 in Taiwan, and TCM physicians can advertise the medical benefits of TCM according to medical law. TCM practitioners are registered TCM physicians with legal licenses who practice in a hospital or clinic. Some TCM physicians holding dual licenses are experts in both Western medicine and TCM due to their college training. These physicians have a high level of expertise and integration in both Western medicine and TCM to provide patients with comprehensive care. Tai Ji and the purchase of non-prescribed CHM were not included in the clinical settings of TCM medical care and they were not covered in the national medical insurance in Taiwan [23, 37]. Although the purchase of non-prescribed CHM is not illegal in Taiwan, TCM physicians and government discourage people to visit CHM store.

Folk therapy in this study included gua-sha (skin scraping), tui-na (massage and kneading), ba-guan (cupping or vacuum bottle therapy), bone setting, spine alignment, qigong, divination, written charms, shaman consultation, talismans, incense ash, and other related practices [22]. The difference between folk therapy and TCM is the legal statuses. Medical law strictly prohibits folk therapy practitioners to declare the medical effects or therapeutic effects in their practice.

In this study, we defined the purchase of non-prescribed CHM as people purchased CHM without physician’s prescription and this phenomenon has been reported in the previous study [23, 37]. Only physician prescribed CHM was considered as part of TCM in this study. Non-prescribed CHM was available in the CHM store but TCM physician do not advice patients to buy or use CHM in the CHM store.

### Statistical analyses

We used chi-square tests to compare the differences in characteristics between older people who did and did not use TCM, including age, sex, marital status, history of disease, smoking, alcohol consumption, emergency care, inpatient care, influenza vaccination, use of folk medicine, and purchase of non-prescribed CHM. The crude odds ratios (ORs) and 95% confidence intervals (CIs) of factors associated with TCM use were calculated by univariate logistic regression. The significant factors (p<0.05) in the univariate analysis were then entered into multivariate logistic regression analysis to calculate the adjusted ORs and 95% CIs of the factors associated with the use of TCM. For each covariate, we assigned a predictive score as a risk index according to the significant adjusted OR, and the predictive score was proportional to the OR. All analyses were performed using Statistical Analysis Software (SAS), version 9.2 (SAS Institute Inc., Cary, North Carolina, USA). A two-sided p value less than 0.05 was considered statistically significant.

## Results

**Fig 1:**
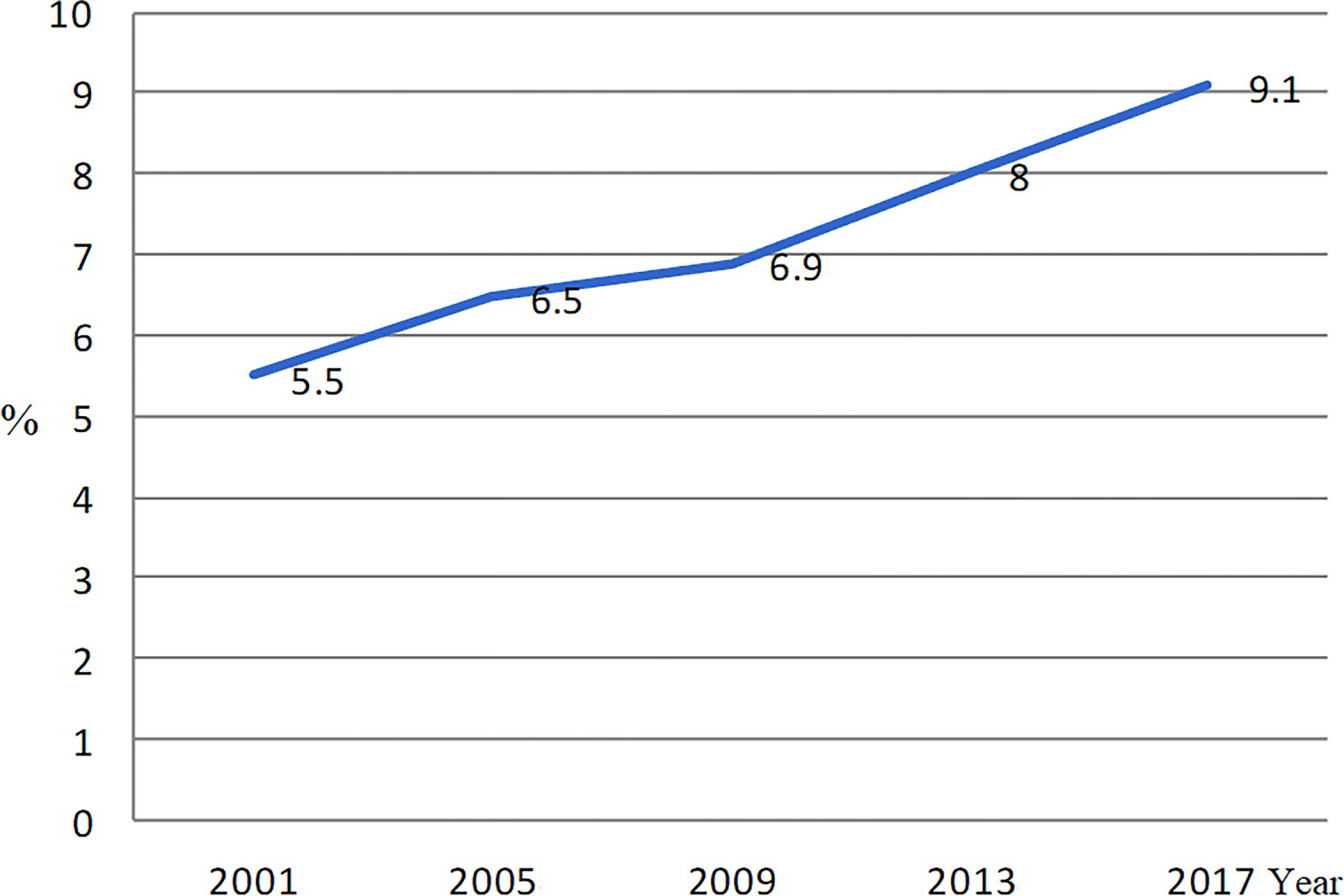
The one-month prevalence of use of traditional Chinese medicine among older people in Taiwan, 2001–2017.

Fig 1 shows that the one-month prevalence of TCM use increased from 5.5% in 2001 to 9.1% in 2017 among older people in Taiwan. Overall, there were 13945 older people in this study, and 7.3% of them had used TCM within the previous month (Table 1). The chi-square test showed that women (8.1%) had a higher use of TCM than men (6.4%, p<0.0001). Higher use of TCM was found among people aged 65–69 years (compared with ≥80 years, p<0.0001) and those who had heart disease (compared with those who did not, p<0.0001), liver disease (compared with those who did not, p = 0.0021), and previous emergency care (compared with those who did not, p = 0.0081). People who smoked (p<0.0001), utilized folk medicine (p<0.0001), and purchase of non-prescribed CHM (p<0.0001) had a higher TCM use than those without these factors.

**Table 1:** The characteristics of older people aged more than 65 years with and without use of traditional Chinese medicine in 2001–2017.

	Use of TCM				p
	No (N = 12926)		Yes (N = 1019)		
Sex	n	(%)	n	(%)	<0.0001
Female	6742	(91.9)	596	(8.1)	
Male	6184	(93.6)	423	(6.4)	
Age, years					<0.0001
65–69	4038	(90.8)	410	(9.2)	
70–74	3335	(92.2)	283	(7.8)	
75–79	2702	(93.7)	181	(6.3)	
≥80	2851	(95.2)	145	(4.8)	
Education					<0.0001
Illiterate	3838	(94.4)	229	(5.6)	
Elementary school	5322	(92.3)	444	(7.7)	
Junior high school	1152	(92.2)	97	(7.8)	
High school	2614	(91.3)	249	(8.7)	
Marital status					0.2980
Married	8033	(92.4)	664	(7.6)	
Unmarried	246	(93.2)	18	(6.8)	
Others	4647		337		
Heart disease					<0.0001
No	10452	(93.2)	767	(6.8)	
Yes	2474	(90.8)	252	(9.2)	
Hypertension					0.5663
No	6704	(92.6)	538	(7.4)	
Yes	6222	(92.8)	481	(7.2)	
Pulmonary disease					0.4307
No	12070	(92.7)	945	(7.3)	
Yes	856	(92.0)	74	(8.0)	
Diabetes					0.8718
No	10311	(92.7)	815	(7.3)	
Yes	2615	(92.8)	204	(7.2)	
Hyperlipidemia					<0.0001
No	9957	(93.2)	728	(6.8)	
Yes	2969	(91.1)	291	(8.9)	
Stroke					0.1382
No	11827	(92.6)	946	(7.4)	
Yes	1099	(93.8)	73	(6.2)	
Liver disease					0.0021
No	12232	(92.9)	941	(7.1)	
Yes	694	(89.9)	78	(10.1)	
Kidney disease					0.2684
No	11838	(92.8)	923	(7.2)	
Yes	1088	(91.9)	96	(8.1)	
Previous hospitalization					0.3337
No	10601	(92.6)	848	(7.4)	
Yes	2325	(93.2)	171	(6.9)	
Previous emergency care					0.0081
No	10425	(93.0)	787	(7.0)	
Yes	2501	(91.5)	232	(8.5)	
Health checkup					0.0007
No	8578	(93.2)	623	(6.8)	
Yes	4348	(91.7)	396	(8.4)	
Influenza vaccination					0.0553
No	6644	(93.1)	492	(6.9)	
Yes	6282	(92.3)	527	(7.7)	
Use of folk therapy					<0.0001
No	9922	(93.4)	701	(6.6)	
Yes	122	(80.8)	29	(19.2)	
Purchasing non-prescribed CHM					<0.0001
No	7223	(93.3)	521	(6.7)	
Yes	592	(87.3)	86	(12.7)	
Smoking					<0.0001
No	11136	(92.3)	931	(7.7)	
Yes	1790	(95.3)	88	(4.7)	
Alcoholic drinking					0.1946
No	12829	(92.7)	1015	(7.3)	
Yes	97	(96.0)	4	(4.0)	
Exercise					0.0191
No	7777	(93.1)	575	(6.9)	
Yes	5149	(92.1)	444	(7.9)	

CHM, Chinese herbal medicine; TCM, traditional Chinese medicine.

As shown in Table 2, age ≥80 years was associated with reduced use of TCM (OR 0.48, 95% CI 0.31–0.72). We also found that people with a history of heart disease (OR 1.62, 95% CI 1.24–2.12), those who used folk medicine (OR 3.16, 95% CI 2.00–4.99), and those who purchased non-prescribed CHM (OR 2.08, 95% CI 1.48–2.91) were more likely to use TCM than those with no heart disease, those who did not use folk medicine, and those who had not purchased non-prescribed CHM. Previous hospitalization was associated with reduced use of TCM (OR 0.59, 95% CI 0.41–0.85).

**Table 2:** Crude and adjusted odds ratios and confidence intervals of factors associated with use of traditional Chinese medicine.

	TCM use (univariate)		TCM use (multivariate)	
	OR	(95% CI)	OR	(95% CI)
Sex				
Female	1.00	(reference)	1.00	(reference)
Male	0.77	(0.68–0.88)	0.78	(0.58–1.04)
Age, years				
65–69	1.00	(reference)	1.00	(reference)
70–74	0.84	(0.71–0.98)	0.87	(0.66–1.15)
75–79	0.66	(0.55–0.79)	0.66	(0.47–0.93)
≥80	0.50	(0.41–0.61)	0.50	(0.33–0.77)
Education				
Illiterate	1.00	(reference)	1.00	(reference)
Elementary school	1.40	(1.19–1.65)	1.28	(0.95–1.71)
Junior high school	1.41	(1.10–1.81)	1.25	(0.78–2.00)
High school	1.60	(1.33–1.92)	1.22	(0.84–1.77)
Marital status				
Married	1.00	(reference)	1.00	(reference)
Unmarried	0.88	(0.55–1.41)	1.26	(0.60–2.65)
Others	0.88	(0.77–1.01)	0.90	(0.68–1.19)
Heart disease				
No	1.00	(reference)	1.00	(reference)
Yes	1.39	(1.20–1.61)	1.61	(1.23–2.10)
Hypertension				
No	1.00	(reference)	1.00	(reference)
Yes	0.96	(0.85–1.10)	0.87	(0.68–1.11)
Lung diease				
No	1.00	(reference)	1.00	(reference)
Yes	1.10	(0.86–1.41)	1.14	(0.80–1.63)
Diabetes				
No	1.00	(reference)	1.00	(reference)
Yes	0.99	(0.84–1.16)	0.93	(0.67–1.28)
Hyperlipidemia				
No	1.00	(reference)	1.00	(reference)
Yes	1.34	(1.16–1.55)	0.92	(0.69–1.23)
Stroke				
No	1.00	(reference)	1.00	(reference)
Yes	0.83	(0.65–1.06)	1.06	(0.68–1.67)
Liver disease				
No	1.00	(reference)	1.00	(reference)
Yes	1.46	(1.15–1.86)	1.32	(0.86–2.03)
Kidney disease				
No	1.00	(reference)	1.00	(reference)
Yes	1.13	(0.91–1.41)	1.44	(1.00–2.07)
Previous hospitalization				
No	1.00	(reference)	1.00	(reference)
Yes	0.92	(0.78–1.09)	0.59	(0.41–0.86)
Previous emergency care				
No	1.00	(reference)	1.00	(reference)
Yes	1.23	(1.06–1.43)	1.32	(0.94–1.85)
Health checkup				
No	1.00	(reference)	1.00	(reference)
Yes	1.25	(1.10–1.43)	1.14	(0.88–1.46)
Influenza vaccination				
No	1.00	(reference)	1.00	(reference)
Yes	1.13	(1.00–1.29)	1.09	(0.86–1.38)
Use of folk therapy				
No	1.00	(reference)	1.00	(reference)
Yes	3.37	(2.23–5.08)	3.14	(1.98–4.97)
Purchasing non-prescribed CHM				
No	1.00	(reference)	1.00	(reference)
Yes	2.01	(1.58–2.57)	2.06	(1.46–2.88)
Smoking				
No	1.00	(reference)	1.00	(reference)
Yes	0.59	(0.47–0.74)	0.85	(0.60–1.22)
Alcoholic drinking				
No	1.00	(reference)	1.00	(reference)
Yes	0.52	(0.19–1.42)	0.56	(0.13–2.33)
Exercise				
No	1.00	(reference)	1.00	(reference)
Yes	1.17	(1.03–1.33)	1.03	(0.81–1.31)

CHM, Chinese herbal medicine; TCM, traditional Chinese medicine.

Table 3 shows the medical visits for TCM among people with various characteristics. Compared with older people without hyperlipidemia, patients with hyperlipidemia had a higher average frequency of TCM use (3.0±3.6 vs. 2.5±2.9 visits, p = 0.0239). Older people who consumed alcohol had fewer visits for TCM use than older people who did not consume alcohol (1.3±0.5 vs. 2.6±3.1 visits, p = 0.0066).

**Table 3:** Visits frequency of traditional Chinese medicine among older people aged more than 65 years in 2001–2017 (N = 1019).

		TCM visits (N = 1019)		
		Mean±SD	β*	P value
Sex	Female	2.6±3.1	0.15	0.4625
	Male	2.7±3.2		
Age, years	65–69	2.7±2.9	0.03	0.8578
	70–74	2.6±3.0		
	75–79	2.6±3.2		
	≥80	2.8±3.7		
Education	Illiterate	2.6±3.7	0.14	0.1174
	Elementary school	2.4±2.5		
	Junior high school	3.0±3.9		
	High school	2.9±3.1		
Marital status	Married	2.6±3.1	0.04	0.8214
	Unmarried	2.4±1.4		
	Others	2.7±3.3		
Heart disease	No	2.6±3.1	0.04	0.8750
	Yes	2.7±3.1		
Hypertension	No	2.5±3.1	0.24	0.2182
	Yes	2.8±3.1		
Lung diease	No	2.6±3.0	0.25	0.6071
	Yes	2.9±4.0		
Diabetes	No	2.6±3.1	0.14	0.5587
	Yes	2.8±3.2		
Hyperlipidemia	No	2.5±2.9	0.54	0.0126
	Yes	3.0±3.6		
Stroke	No	2.6±3.0	1.01	0.0641
	Yes	3.6±4.5		
Liver disease	No	2.6±3.0	0.70	0.1800
	Yes	3.3±4.5		
Kidney disease	No	2.7±3.2	-0.54	0.1052
	Yes	2.1±1.6		
Hospitalization	No	2.7±3.1	-0.13	0.6141
	Yes	2.5±3.2		
Emergency care	No	2.6±3.1	0.03	0.8957
	Yes	2.7±3.2		
Health checkup	No	2.6±3.0	0.09	0.6623
	Yes	2.7±3.3		
Influenza vaccination	No	2.6±3.0	0.15	0.4445
	Yes	2.7±3.3		
Use of folk therapy	No	2.6±3.2	0.95	0.3011
	Yes	3.6±4.8		
Purchasing non-prescribed CHM	No	2.6±3.2	0.31	0.5103
	Yes	2.9±4.2		
Smoking	No	2.6±3.1	-0.06	0.8591
	Yes	2.6±2.7		
Alcoholic drinking	No	2.6±3.1	-1.39	0.0066
	Yes	1.3±0.5		
Exercise	No	2.5±2.6	0.31	0.1152
	Yes	2.8±3.6		

CHM, Chinese herbal medicine; TCM, traditional Chinese medicine.

*Multiple regression analysis for the factors associated with use frequency of TCM.

## Discussion

Using data from the National Health Interview Survey in Taiwan, we found a high prevalence rate of 7.3% for the utilization of TCM within the past month in the population older than 65 years in 2001–2017. Age, cardiovascular disease, kidney disease, the use of folk medicine and the purchase of non-prescribed CHM were associated with the long-term utilization of TCM.

Among Chinese-American older adults in Chicago, 76% reported any use of TCM within the past year [10]. The lifetime prevalence of the use of TCM among people aged 50 years and older in Shanghai was 50% [12]. Comparing the use of TCM in the Chinese population in other countries [10, 12], the corresponding rate of TCM use in this study was relatively low (7.3% within the past month). A possible explanation is that the contents of TCM use in other reports in America or China included treatment procedures not performed by physicians. Certainly, the estimated period of utilization varied in reports and is also a reason for the discrepancy in TCM use.

The utilization of TCM among older people in Taiwan has been reported in previous studies [6, 14, 20]. In 2001, the one-year prevalence of TCM use among people aged 65 and over in Taiwan was 25.9% [6]. A national health survey in Taiwan showed that the prevalence of TCM use among older people aged over 70 years in 2002 was 7.7% [14]. From 2005–2009, 48% (the five-year prevalence) of older people had used TCM at least once in Taiwan [20]. The use of TCM in our study was higher than that in a previous report because we measured utilization among elderly individuals on a monthly basis instead of annually, and it is important to note the differences in utilization rates measured over different time periods. In Taiwan, both the ratio of TCM users and mean TCM visits increased gradually from 2000 to 2005 and even further to 2010 among elderly people [24]. However, we could not exclude the possibility of recall bias among the older people in this study.

Our study found that older people aged 75–79 or ≥80 years had lower ORs of TCM utilization than those aged 65–69 years. The findings of this study were consistent with those of previous studies in Taiwan that indicated that younger people possessed much more knowledge, adequate attitudes, and active practices regarding TCM and exhibited a higher frequency of TCM utilization than older people [14, 20, 25]. Moreover, living in health care institutions, limited mobility, and regular visits to Western medicine practitioners (such as for renal dialysis) are possible reasons why older people aged 75 and above had a reduced use of TCM in this study.

A study based on a systematic review suggested the beneficial effects of TCM on cardiovascular disease, hypertension, and the outcomes of coronary heart disease and heart failure [26]. Dan Shen is a common herb in CHM that is widely used in Asia because of its benefits for cardiovascular diseases [27]. It contains hydrophilic phenolic acids and lipophilic tanshinones, which are believed to contribute to its therapeutic efficacy. Studies have shown that Dan Shen reduces the risk of coronary heart disease in patients and protects the vascular endothelium [28, 29]. Moreover, previous studies also identified Dan Shen as a commonly prescribed TCM prescription for elderly individuals in Taiwan [21]. Therefore, it is reasonable that older people with heart disease were more likely to use TCM than those with no heart disease in this study.

Taiwan has the highest incidence of end-stage renal disease worldwide [30]. The risk of developing chronic kidney disease shows an exponential increase after 60–64 years [31]. Due to the limited treatment options for chronic kidney disease in clinical conventional medicine settings, some patients turn to secondary medical advice or alternative therapies, including TCM [32]. TCM has shown potential beneficial effects on improving kidney function, hemoglobin levels, and serum albumin levels and reducing proteinuria [33–36]. However, we found that older people with kidney disease had a higher likelihood of using TCM but had a lower frequency of TCM use in this study. This implies that older people with chronic kidney disease used TCM once. We considered that patients with end-stage renal disease receiving dialysis two or three times weekly might have had less opportunity to use TCM.

In this study, we found that older people who used folk medicine had a higher likelihood of TCM use, which is consistent with the results of previous studies [22]. In Taiwan, TCM includes CHM, acupuncture, moxibustion, bone reduction, traditional trauma treatment, traditional dislocation treatment, traditional fracture treatment, tui-na, ba-guan, and other therapies. Folk medicine also includes acupressure, Chinese massage, cupping and skin scraping, and CHM without a physician’s prescription. Unsurprisingly, the use of folk medicine was associated with the utilization of TCM in this study because several items and contents are similar between folk medicine and TCM. We also considered that older people may not recognize the safety, legality, or efficacy of TCM or folk medicine. In fact, TCM practitioners in Taiwan are registered TCM physicians who are certified, licensed, and practice in a hospital or clinic setting. TCM in Taiwan is recognized as a health service and treatment modality, and TCM physicians can legally advertise the medical benefits of TCM and claim reimbursement from National Health Insurance.

Purchasing CHM without a physician’s prescription is common in Taiwan [23, 37]. In this study, we found that purchasing non-prescribed CHM was associated with the use of TCM among older people. A previous study found that older people purchased non-prescribed CHM more than other age groups [23]. Another study also suggested that TCM outpatient visits with the purchase of non-prescribed TCM products (not covered by National Health Insurance) were more frequent among individuals over the age of 65 compared to other age groups [38]. With the widespread presence of Chinese herbal pharmacies and the possibility of purchasing most types of non-prescribed CHM, there is a noticeable trend indicating a heightened acceptance among people toward the use and purchase of non-prescribed CHM in Taiwan. Physicians need to understand the conditions regarding the purchase of non-prescribed CHM among their patients.

The previous two studies had found that use of folk therapy was significantly associated with the purchase of non-prescribed CHM [22, 23, 37]. We could hypothesize that people who choose to use folk therapy or to purchase non-prescribed CHM were the active population with seeking second treatment opinion. Many patients choose to use folk therapy or purchase of non-prescribed CHM based on a recommendation from someone who has used the therapy and has been satisfied with the results [22, 23, 37]. However, the health authority and physicians discouraged patients to seek folk therapy or to purchase non-prescribed CHM. It should be cautioned that non-prescribed CHM and folk therapy per se and the interactions within TCM, non-prescribed CHM, and folk therapy were risky because the uncertain content and unlicensed executors of non-prescribed CHM and folk therapy.

There were some limitations that should be noted when interpreting the results of this study. First, there was a possibility of recall bias among the elderly participants, as their ability to accurately recall past health care experiences may have been influenced by various factors. Because the information of sociodemographic factors, medical conditions and utilization were defined as within recent 12 months before the face-to-face questionnaire interview, the recall bias could not be excluded in this study, Second, the cross-sectional study design used in this research did not allow us to establish causal relationships between the use of TCM and related factors. Therefore, caution should be exercised when interpreting the findings of this study in terms of causality. Third, it is important to acknowledge that the timeframe of the analyses spanned a wide range. The potential effects of temporal and policy influences on the utilization of TCM could not be controlled for in this study. Fourth, other factors have been reported as factors associated with TCM use that could not be considered in this study, such as socioeconomic status, physical activity or exercise, and family support. Although we used multiple logistic regression and controlled for many potential confounding factors, residual confounding may persist in this study.

In conclusion, the use of TCM increased in 2001–2017 among older population aged 65 and older in Taiwan and the one-month prevalence of TCM use was associated with age, cardiovascular disease, kidney diseases, use of folk medicine, and the purchase of non-prescribed CHM. This study highlights the need for health care among older people who seek TCM and could be considered a reference for health regulatory agencies in future policies.

## List of abbreviations

CI: confidence interval
OR: odds ratio
TCM: traditional Chinese medicine
